# Investigation of SP94 Peptide as a Specific Probe for Hepatocellular Carcinoma Imaging and Therapy

**DOI:** 10.1038/srep33511

**Published:** 2016-09-21

**Authors:** Yanli Li, Yan Hu, Jie Xiao, Guobing Liu, Xiao Li, Yanzhao Zhao, Hui Tan, Hongcheng Shi, Dengfeng Cheng

**Affiliations:** 1 Department of Nuclear Medicine, Zhongshan Hospital, Fudan University, Shanghai 200032, China; 2 Shanghai Institute of Medical Imaging, Shanghai 200032, China; 3 Institute of Nuclear Medicine, Fudan University, Shanghai 200032, China; 4 Jining NO.1 People’s Hospital, Jining, Shandong 272000, China

## Abstract

SP94 (SFSIIHTPILPL), a novel peptide, has shown specific binding to hepatocellular carcinoma (HCC) cells. We aimed to investigate the capability of SP94 as a targeting probe for HCC imaging and therapy following labeling with technetium-99m (^99m^Tc) and rhenium-188 (^188^Re). HYNIC-SP94 was prepared by solid phase synthesis and then labeled with ^99m^Tc. Cell competitive binding, internalization assay, *in vitro and in vivo* stability, biodistribution and micro-single photon emission computed tomography /computed tomography (SPECT/CT) imaging studies were performed to investigate the capability of ^99m^Tc tricine-EDDA/HYNIC-SP94 as a specific HCC imaging probe. Initial promising targeting results inspired evaluation of its therapeutic effect when labeled by ^188^Re. HYNIC-SP94 was then labeled again with ^188^Re to perform cell apoptosis, microSPECT/CT imaging evaluation and immunohistochemistry. Huh-7 cells exhibited typical apoptotic changes after ^188^Re irradiation. According to ^99m^Tc tricine-EDDA/HYNIC-SP94 microSPECT/CT imaging, tumor uptake was significantly decreased compared with that of pre-treatment with ^188^Re-HYNIC-SP94. The immunohistochemistry also displayed obvious necrosis and apoptosis as well as inhibition of proliferation in the ^188^Re-HYNIC-SP94 treatment group. The results supported that ^99m^Tc tricine-EDDA/HYNIC-SP94 is able to target HCC cells and ^188^Re-HYNIC- SP94 holds potential as a therapeutic agent for HCC, making ^99m^Tc/^188^Re-HYNIC-SP94 a promising targeting probe for HCC imaging and therapy.

Hepatocellular carcinoma (HCC) is the most common primary malignancy (70~85%) of the liver. Worldwide, it is the fifth most common cancer in men (522 400 cases, 7.9% of all cancers), the seventh most common in women (225 900 cases, 3.7% of all cancers) and the third most common cause of cancer-related death^1^,^2^,^3^. Moreover, the median survival for most HCC patients is estimated to be 1 year^4^,^5^. Early diagnosis together with prompt surgical removal and other combined therapeutic strategies offer patients the best opportunity for treatment or prolonged survival. Thus, the development of specific radiopharmaceuticals for HCC imaging and therapy is of great interest due to high specificity and sensitivity of nuclear medicine.

Radionuclide imaging with single photon emission computed tomography (SPECT) or positron emission tomography (PET) is valuable as a functional imaging modality for the diagnosis and staging of HCC. Furthermore, the use of radiolabeled peptides as probes for molecular imaging and radioimmunotherapy has been encouraging^6^. We developed the novel ^99m^Tc/^188^Re-labeled SP94 peptide analog, designated as ^99m^Tc/^188^Re-HYNIC-SP94, and investigated its potency for HCC imaging and therapy in this study.

SP94 (SFSIIHTPILPL), a novel peptide identified by Lo *et al*.^7^ using a phage display technique, can specifically bind to various types of HCC cells, such as HepG2 and Huh-7^8^,^9^. HCC cell membrane expresses an unknown target molecule that can be recognized by SP94 but not by hepatocytes. Ashley *et al*.^10^ conjugated SP94 with nanoparticle-supported lipid bilayers to improve therapeutic efficacy and reduce side effects, and it was found to be bound to HCC cells with high affinity owing to the recruitment of SP94 targeting peptides. Their further results indicated that SP94 modified with MS2 virus-like particles exhibited a 10^4^-fold higher affinity for HCC than for hepatocytes^11^.

Since SP94 can specifically bind to HCC, we hypothesized that it could be used as a probe for molecular imaging and even for radioimmunotherapy, after being labeled with a suitable radionuclide such as ^99m^Tc or ^188^Re. ^99m^Tc labeling remains a striking approach for peptide SPECT/CT imaging because of its 140 keV γ-photon emission and a manageable 6 h half-life. Additionally, ^99m^Tc is highly cost-effective and can be easily obtained from a commercial ^99^Mo-^99m^Tc generator^12^,^13^,^14^,^15^. ^188^Re is an attractive radionuclide for targeted radiotherapy and imaging because of its optimal physical characteristics of high-energy *β*-particles (E_max_ = 2.12 MeV) and γ-photons (155-keV) in 15% abundance^16^,^17^. In addition, strong tissue penetration effects (max 11 mm, mean range 3.5 mm), suitable half-life (16.9 hours) and convenient availability from a generator have greatly increased the research and clinical application of ^188^Re-labeled radiopharmaceuticals^18^,^19^.

Although SP94 has been proposed as a targeting peptide to deliver drugs to treat advanced HCC^8^,^10^,^20^, SP94 as an imaging and therapeutic agent for HCC using the same pharmacophore but different isotopes has not been investigated so far. Therefore, in this study, SP94 was radiolabeled with ^99m^Tc and ^188^Re, and *in vitro* and *in vivo* studies were performed to evaluate its capability as a specific probe for HCC imaging and therapy.

## Results

### Results for ^99m^Tc tricine-EDDA/HYNIC-SP94

**Figure 1:**
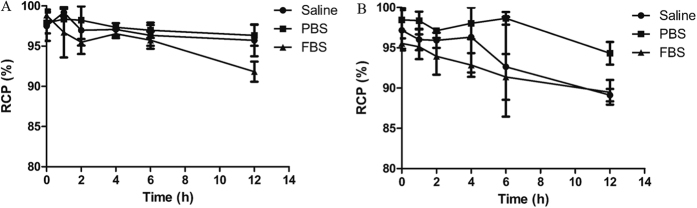
The stability curves of the ^99m^Tc-labeled complexes in normal saline, PBS and FBS. (**A**) Using tricine/EDDA as coligand. (**B**) Using tricine as coligand.

**Radiochemistry and stability.** HYNIC-SP94 could be labeled at high specific activities (30 GBq/μmol) using tricine or tricine/EDDA as coligands. The average labeling yields were >95% for ^99m^Tc tricine/HYNIC-SP94 and ^99m^Tc tricine-EDDA/HYNIC-SP94. The stability experiment in phosphate buffer (PBS), normal saline and fetal bovine serum (FBS) revealed high stabilities of ^99m^Tc-HYNIC- SP94 irrespective of which coligand was used. However, the stability of ^99m^Tc tricine-EDDA/HYNIC-SP94 was slightly better than that of ^99m^Tc tricine/HYNIC-SP94 (Fig. 1); therefore, ^99m^Tc tricine-EDDA/HYNIC-SP94 was used for further evaluation.

### *In vitro* HCC cell competitive receptor binding assay

**Figure 2:**
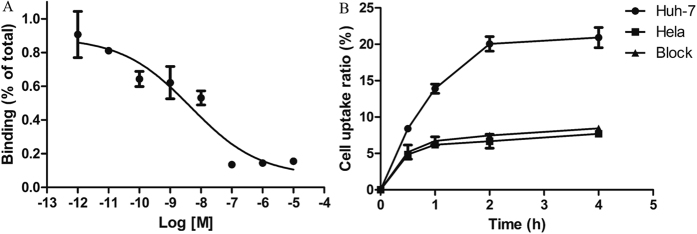
Displacement curve and cell uptake study of ^99m^Tc tricine-EDDA/HYNIC-SP94. (**A)** The IC_50_ value was calculated to be 4.49 ± 0.20 nM. Log [M] = log of increasing concentration (mol/L) of HYNIC-SP94. (**B)** Uptakes of ^99m^Tc tricine-EDDA/HYNIC-SP94 in Huh-7 and Hela cells were measured at the indicated time points.

Results of a comparative displacement assay in Huh-7 cells were plotted as a sigmoid curve for the displacement of ^99m^Tc tricine-EDDA/HYNIC-SP94 when concentrations of HYNIC-SP94 increased (from 0.001 nM to 10000 nM). The binding rate was plotted as percentage binding of total activity added. The IC_50_ value was 4.49 ± 0.20 nM, which indicated that SP94 peptide showed a high binding affinity on Huh-7 cell membranes. An example of the competitive binding curve is shown in Fig. 2A.

### Internalization assay

The uptake rates of ^99m^Tc tricine-EDDA/HYNIC-SP94 by Huh-7 and Hela cells were determined (Fig. 2B). At 0.5 h after incubation of ^99m^Tc tricine-EDDA/HYNIC-SP94 with Huh-7 cells, the uptake rate was 8.41 ± 0.31% and increased to 20.92 ± 1.38% at 4 h. However, Hela cells showed a significantly lower uptake rate of only 7.69 ± 0.03% at 4 h (P < 0.001). In the blocking study performed in Huh-7 cells, the tracer uptake rate was 8.46 ± 0.22% at 4 h, which was approximately 2.5-fold less than that of the non-blocked group. Indeed, statistical analysis revealed that there was a significant difference in uptake between the unblocked and blocked groups (P < 0.001). These data suggested that ^99m^Tc tricine-EDDA/HYNIC-SP94 specifically binds to and penetrates the Huh-7 cell membranes.

### *In vivo* stability

It was found that ^99m^Tc tricine-EDDA/HYNIC-SP94 could maintain about 66% of the radiochemical purity (RCP) within 60 min, which indicated that it has a suitable *in vivo* stability regarding tumor targeting. Furthermore, no sign of free ^99m^Tc release was found in the following biodistribution study based on low radioactivity uptakes in stomach and thyroid. With comprehensive analyses of blood samples using reversed-phase high-performance liquid chromatography (RP-HPLC) and biodistribution, favorable *in vivo* stability of ^99m^Tc tricine-EDDA/HYNIC-SP94 was demonstrated.

### Biodistribution

Biodistribution of ^99m^Tc tricine-EDDA/HYNIC-SP94 was evaluated in Huh-7 tumor-bearing mice. Results are shown in Table 1 as the percentage of injected dose per gram of tissue (%ID/g). Uptake in the kidneys was highest (7.40 ± 2.66% ID/g) because radioactivity was rapidly cleared from the blood via the preferred renal-urinary route. Aside from the bladder and kidneys, labeled peptide showed moderate uptake values in the Huh-7 tumors, which was higher at 0.5 h (1.02 ± 0.26% ID/g) than at 1.5 h post-injection (p.i.) (0.74 ± 0.07% ID/g). The tumor uptake only reduced significantly (0.34 ± 0.09% ID/g; P = 0.024) after injection of the blocking agent, indicating specific receptor targeting of ^99m^Tc tricine-EDDA/HYNIC-SP94.

### MicroSPECT/CT imaging

**Figure 3:**
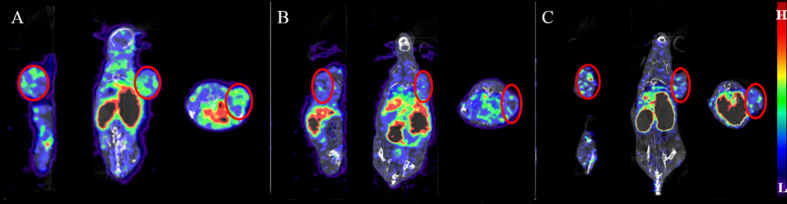
Small-animal SPECT/CT imaging in the tumor-bearing model. MicroSPECT/CT images of ^99m^Tc tricine-EDDA/HYNIC-SP94 in Huh-7 tumor-bearing mice at 0.5 h p.i. without blocking agent (**A**) and with blocking agent (**C**) as well as in Hela tumor-bearing mice (**B**). Circles indicate the tumors.

Figure 3 shows microSPECT/CT images at 0.5 h after injection of ^99m^Tc tricine-EDDA/HYNIC-SP94 with or without the blocking agent in Huh-7 and Hela tumor-bearing mice. The Huh-7 tumor could be seen clearly at 0.5 h after injection of ^99m^Tc tricine-EDDA/HYNIC-SP94 (Fig. 3A), while no radioactivity accumulation was observed in the Hela tumors at the same time point (Fig. 3B). In addition, the tumor uptake was not visualized in the blocked mice (Fig. 3C).

### Results for ^188^Re-HYNIC-SP94

**Figure 4:**
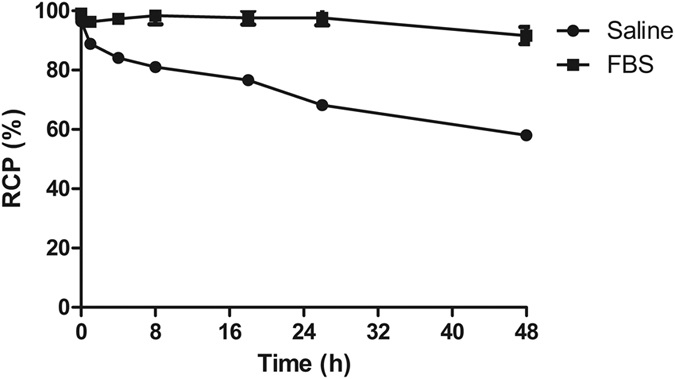
Stability assay. *In vitro* stability test of ^188^Re-HYNIC-SP94 at specific times after incubation in normal saline and FBS at room temperature.

**Radiolabeling and stability of ^188^Re-HYNIC-SP94.** The RCP and the specific activity of ^188^Re-HYNIC-SP94 were 96% and 1.2 GBq/μmol, respectively. The *in vitro* stability studies showed that when incubated in normal saline and FBS, the RCP of ^188^Re-HYNIC-SP94 dropped to 84.17 ± 0.01% and 97.27 ± 0.25% at 4 h, respectively, but still remained at 91.67 ± 2.36% until 48 h, in FBS (Fig. 4).

#### Detection of apoptosis induction by ^188^Re-HYNIC-SP94 in Huh-7 and Hela cells

**Figure 5:**
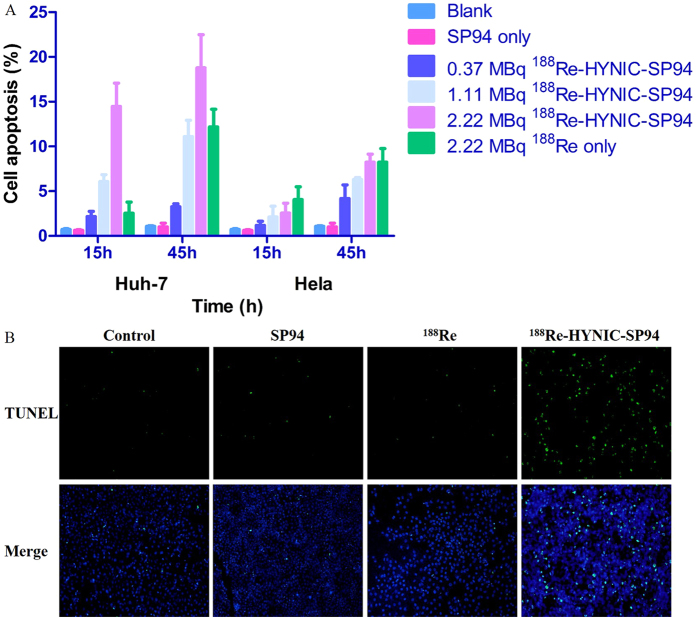
Comparison of different conditions induced apoptosis in Huh-7 and Hela cells by TUNEL assay. (**A)** Quantitative analysis of apoptosis in Huh-7 and Hela cells. (**B)** Images of captured apoptotic cells by a fluorescence microscope; the green-fluorescent signal represented cells that underwent apoptosis and DAPI was used for a nuclear stain.

TUNEL assay was performed to detect apoptotic cells. After being incubated with ^188^Re-HYNIC-SP94, ^188^Re, SP94 and medium alone, the cells were observed with a fluorescent microscope, and green signals indicated apoptotic cells. The data indicated that cell apoptosis induced by ^188^Re-HYNIC-SP94 and ^188^Re was dose- and time-dependent in the two cell lines, especially in Huh-7 cell line treated with ^188^Re-HYNIC-SP94. Huh-7 cell apoptosis increased from nearly 2.16 ± 0.46% at 0.37 MBq to 14.46 ± 2.14% at 2.22 MBq in the ^188^Re-HYNIC-SP94 treatment group at 15 h (P = 0.001). In comparison, 2.22 MBq of ^188^Re only induced 2.54 ± 1.01% cell apoptosis (P = 0.002). Increasing the incubation time of ^188^Re-HYNIC-SP94 (1.11 MBq) from 15 to 45 h caused Huh-7 cell apoptosis from 6.07 ± 0.63% to 11.10 ± 1.50% (P = 0.012). In addition, in the SP94, blank and Hela groups, no obvious apoptotic cells were observed (Fig. 5A). As seen in Fig. 5B, ^188^Re-HYNIC-SP94 treatment significantly increased apoptosis of Huh-7 cells when compared with the ^188^Re, blank and SP94 treatment groups. These results all indicated that SP94 can target Huh-7 cells and induce apoptosis by virtue of radiation from labeled ^188^Re.

#### Evaluation of therapeutic efficacy induced by ^188^Re-HYNIC-SP94 using ^99m^Tc tricine-EDDA/HYNIC-SP94 microSPECT/CT imaging

**Figure 6:**
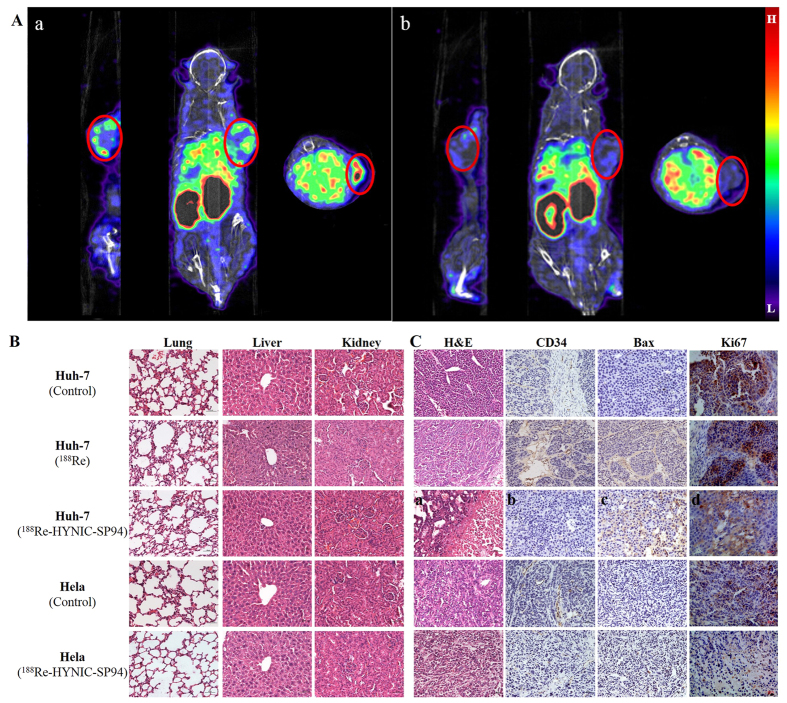
Evaluation of therapeutic efficacy induced by ^188^Re-HYNIC-SP94. (**A)** MicroSPECT/CT images of Huh-7 tumor-bearing mice before (a) and after (b) intravenous injection of ^188^Re-HYNIC-SP94. Tumor uptakes were visualized as indicated by circles. (**B)** H&E staining of lungs, liver and kidneys before and after treatment with ^188^Re-HYNIC-SP94. No significant morphological damage occurred in ^188^Re-HYNIC-SP94 treated and control groups. (**C)** H&E staining and immunohisto-chemistry for CD34, bax and Ki67 in tumors. ^188^Re-HYNIC-SP94 treatments led to extensive necrosis (a), little expression of CD34 (b, brown color), strong expression of bax (c, brown color) and weak expression of Ki67 (d, brown color) in Huh-7 tumors. The scale bar represents 50 μm and magnification is 200x.

To confirm the therapeutic efficacy of ^188^Re-HYNIC-SP94, microSPECT/CT imaging was performed. The microSPECT/CT images of Huh-7 tumor-bearing mice before and after injection of ^188^Re-HYNIC-SP94 are presented in Fig. 6A. Significant uptake of ^99m^Tc tricine-EDDA/HYNIC-SP94 was visualized in the Huh-7 tumor before injection of ^188^Re-HYNIC-SP94, but no accumulation of ^99m^Tc tricine-EDDA/HYNIC-SP94 was observed at 24 h after injection of ^188^Re-HYNIC-SP94. The results demonstrated that the tumor uptake of ^99m^Tc tricine-EDDA/HYNIC-SP94 was partially inhibited by ^188^Re-HYNIC-SP94 via inducing cell apoptosis to decrease the specific receptors of HCC cell membranes.

#### Hematoxylin and eosin (H&E) staining and immunohistochemistry

For major organs, such as lungs, liver and kidneys, no macroscopic abnormalities and histopathological damage were observed in any of the irradiated and control mice, suggesting no toxicity in the treated mice (Fig. 6B). CD34 is a transmembrane glycoprotein on cells, which can promote the aggregation of endothelial progenitor cells to induce angiogenesis and can be used to evaluate the risk of recurrence and prognosis. After treatment with ^188^Re-HYNIC-SP94, the amount of newly generated vessels was significantly reduced when compared with the other groups, indicating that ^188^Re-HYNIC-SP94 effectively inhibited the invasion and metastasis of cancer cells. Bax is a pro-apoptotic protein and was highly expressed only in Huh-7 tumor tissue after treatment with ^188^Re-HYNIC-SP94, which showed as yellow-brown or brown tissue (Fig. 6C). The expression of bax can be used to evaluate the extent of apoptosis, which reflects the therapeutic efficacy of ^188^Re-HYNIC-SP94. Ki-67 is a nuclear antigen that only exists in proliferating cells and is widely used as a marker of cell proliferation. Ki-67 expression in ^188^Re-HYNIC-SP94-treated Huh-7 tumor tissue was lower (12.33 ± 0.02%) than that of the control group (41.33 ± 0.04%) (P = 0.001) and the ^188^Re treated group (30.67 ± 0.03%) (P = 0.001), suggesting that ^188^Re-HYNIC-SP94 could inhibit the excessive proliferation of HCC cells.

## Discussion

SP94 peptide, comprising twelve amino acids, can selectively bind to HCC cells and is under development as a targeting peptide for therapeutic purposes^8^,^10^,^20^. Recently, polymer coupled with SP94 peptide was found to exhibit a 10^4^-fold greater avidity for HCC than for hepatocytes^10^,^11^. Based on these results, SP94 may be a good candidate as an imaging or therapeutic probe to detect HCC, when labeled with ^99m^Tc or ^188^Re.

There are many bifunctional chelating agents (such as HYNIC and NHS-MAG3) that could be used for both ^99m^Tc and ^188^Re labeling of biomolecules. Among them, HYNIC has demonstrated a number of advantages such as high RCP and good stability, as well as a predominantly renal excretion^21^. Because HYNIC can only occupy one or two coordination positions on the radionuclide, coligands (such as tricine and EDDA) are necessary to complete the radionuclide coordination sphere^22^,^23^. Decristoforo *et al*.^24^,^25^ have used EDDA as a coligand, showing that it has advantages over other coligands in terms of peptide labeling. However, Erfani *et al*.^26^ found that in a group of different coligands, tricine offered the best radiolabeling efficiency. In this study, we tested two coligand systems using tricine and tricine/EDDA. The differences between the two coligand systems did not appear very pronounced; however, because of its better stability, ^99m^Tc tricine-EDDA/HYNIC-SP94 was selected for further evaluation. ^99m^Tc tricine-EDDA/HYNIC-SP94 was synthesized with high radiochemical yield (>96%) and proved highly stable in normal saline, FBS and PBS (Fig. 1).

The IC_50_ value for HYNIC-SP94 was 4.49 ± 0.20 nM from competitive binding assay (Fig. 2A), which suggested that SP94 peptide had a high binding affinity on Huh-7 cells. Percentage of internalization was around 20% for Huh-7 cells (Fig. 2B), indicating that SP94 peptide has the ability to penetrate the HCC cell membranes. Based on *in vivo* stability assay, the labeled peptide could maintain about 66% of the RCP within 60 min, indicating a fair stability according to its *in vivo* tumor targeting and pharmacokinetics. *In vivo* biodistribution experiments showed rapid tumor uptake, higher at 0.5 h (1.02 ± 0.26% ID/g) and dropping slightly at 1.5 h (0.74 ± 0.07% ID/g) p.i. ^99m^Tc tricine-EDDA/HYNIC-SP94 uptake was highest in the kidneys but ^99m^Tc tricine-EDDA/HYNIC-SP94 was rapidly cleared from the blood via the preferred renal-urinary route, thus the retention time was quite short, owing to the kidney being the major organ for metabolism. Pre-injection with the blocking agent did not reduce kidney uptake or excretion but significantly decreased tumor uptake of ^99m^Tc tricine-EDDA/HYNIC-SP94. In other organs such as liver and lungs, a moderate radioactivity accumulation was observed, possibly caused by the saturation effect of SP94 in the distribution mechanisms (Table 1). Using SPECT/CT imaging, Huh-7 tumors were clearly delineated from normal tissues as early as 0.5 h after injection of ^99m^Tc tricine-EDDA/HYNIC-SP94 (Fig. 3A), but no obvious tracer could be seen in Hela tumors (Fig. 3B) and tumor uptake of the radiolabeled peptide was significantly reduced (P = 0.024) in the blocked group (Fig. 3C). The moderate uptake of ^99m^Tc tricine-EDDA/HYNIC-SP94 for the liver was due to the lipophilicity of this tracer; however, concentration differences in radioactivity enabled the tumor area to be distinguished from the liver. Imaging and biodistribution studies further revealed that ^99m^Tc tricine-EDDA/HYNIC-SP94 exhibited clear targeting of HCC.

The current major limitation to use this peptide is its fast metabolism in plasma by endogenous peptidases under physiological conditions^27^. Also, background interference from the liver may hinder the detection of tumors to some degree. However, as discussed earlier, when SP94 peptide is conjugated to polymers, its *in vivo* behavior is improved, encouraging us to make further modification to this peptide^10^,^11^. Moreover, *in vitro* and *in vivo* results all supported that ^99m^Tc tricine-EDDA/HYNIC-SP94 can target HCC cells and this finding may have potential application in the design of an internal radiotherapy agent for HCC, when the peptide was labeled again with ^188^Re.

During the past decades, radiolabeled peptides have been investigated as potential therapeutic agents, both in research and in clinic; for example, ^188^Re-HYNIC-trastuzumab enhanced the effect of apoptosis in HER2-overexpressing breast cancer cells and ^188^Re-labeled HEDP could be used for internal radiation therapy to treat painful bone metastases^28^,^29^. The *β* ray (mean range 3.5 mm) emitted by ^188^Re causes little injury to surrounding tissues and can induce apoptosis for the internal radiation therapy of tumors. In this study, we revealed that ^188^Re-HYNIC-SP94 led specifically to apoptosis of HCC cells, in a radioactivity dose- and time-dependent manner. The data suggested that ^188^Re-HYNIC- SP94 has the potential for use as a therapeutic radiopharmaceutical in HCC treatment.

In this study, ^188^Re-HYNIC-SP94 could be labeled with 96% of RCP and remained at a level >90% after 48 h of incubation with FBS (Fig. 4); these properties suggested its therapeutic function was worthy of further evaluation. TUNEL staining showed significant apoptosis of Huh-7 cells after ^188^Re-HYNIC-SP94 treatment compared with the control groups (Fig. 5). This may has been due to the deep penetration of SP94 peptide and irradiation on the tumor cells by the energetic *β-*emissions^30^. The ^99m^Tc tricine-EDDA/HYNIC-SP94 microSPECT/CT images of Huh-7 tumor-bearing mice showed significant differences in tumor uptake before and after injection of ^188^Re-HYNIC-SP94 (Fig. 6A), revealing the loss of specific receptors in the cell membranes because of cell apoptosis induced by radiation from ^188^Re. This indicated that ^188^Re-HYNIC-SP94 is a favorable therapeutic agent in HCC treatment. The above results were consistent with H&E staining and immunohistochemistry analysis of bax, which showed massive necrosis and apoptosis in Huh-7 tumors but not in Hela tumors, after treatment by ^188^Re-HYNIC-SP94. The immunohistochemistry analysis of CD34 showed the suppression of angiogenesis after ^188^Re-HYNIC-SP94 treatment. Ki-67 expression in ^188^Re-HYNIC-SP94-treated Huh-7 tumor tissue was lower than that of other treatment groups (Fig. 6C). These findings demonstrated that ^188^Re-HYNIC-SP94 can induce cell apoptosis and inhibit excessive HCC cell proliferation, providing evidence of its potential for the internal radiotherapy of HCC. Although tumor size changes were not observed in this study, it was interesting to find apparent tumor receptor and pathological changes from imaging and other *in vitro* assays. Furthermore, it was observed from H&E staining and immunohistochemistry analyses of normal organs such as kidneys, liver and lungs, together with the biodistribution studies, that no further damage was observed on the main organs aside from the tumors.

Although some small molecules and peptides labeled by ^188^Re have been shown to posses the capacity for treatment of HCC, our imaging and therapeutic probes (^99m^Tc/^188^Re-HYNIC-SP94) still have potential applications, besides commercial availability, easy labeling, tumor specificity, fast clearance *et al*., most important is that HYNIC-SP94 could be served as an hybrid imaging and therapeutic agent for HCC by using of the same pharmacophore but different isotopes. Therefore, it will be meaningful that SP94 was radiolabeled with ^99m^Tc and ^188^Re to evaluate its capability as a specific probe for HCC imaging and therapy.

## Conclusion

*In vitro* and *in vivo* results all supported that ^99m^Tc tricine-EDDA/HYNIC-SP94 can target HCC cells. However, its *in vivo* behavior requires further modification, by conjugating the peptide with other carriers such as nanoparticles or polymers to improve tumor targeting and pharmacokinetics. Nevertheless, its high specificity to HCC cells warrants its potential as a HCC radiotherapeutic agent when labeled with ^188^Re. TUNEL assay, SPECT/CT imaging and immunohistochemistry results revealed that ^188^Re-HYNIC-SP94 has the potential as a therapeutic radiopharmaceutical agent in HCC treatment. The inhibitory effects in Huh-7 cells suggested that ^188^Re-HYNIC-SP94 may be therapeutically beneficial for HCC patients in the future. When labeled with ^99m^Tc or ^188^Re, HYNIC-SP94 has the potential for using as a diagnostic or therapeutic agent for HCC.

## Methods

### Materials and instruments

6-hydrazinonicotinic acid-SP94 (HYNIC-SP94) with a purity of 95% was purchased from China Peptides Co, Ltd (Hangzhou, China). ^99m^TcO_4_^−^ was provided by Shanghai Atom Kexing Pharmaceutical Co, Ltd (Shanghai, China) and carrier-free ^188^Re was supplied by Jiangsu Laitai Medical Biotechnology Co, Ltd (Jiangsu, China). Tricine and SnCl_2_·2H_2_O were purchased from Sigma-Aldrich Corporation (St. Louis, USA); ethylenediamine-N,N′-diacetic acid (EDDA) and glucohepatonate (GH) were supplied by TCI Corporation (Tokyo, Japan). All other reagents were provided by China National Pharmaceutical Group Corporation (Beijing, China). *In Situ* Cell Death Detection Kit was purchased from Roche Applied Science (Mannheim, Germany). The Sep-Pak C_18_ columns were supplied by Waters Corporation (Milford, USA). The following instruments were used: γ-counter (GC-1200, Zhongcheng, Hefei, China); RP-HPLC (Agilent Technologies, Santa Clara, USA); radio-thin layer chromatography (Radio-TLC, Bioscan InC, Washington DC, USA); MicroSPECT/CT (NanoSPECT/CT, Bioscan InC, Washington DC, USA); fluorescence microscope (DLMB2, Leica, Wetzlar, Germany).

Human hepatocellular carcinoma (Huh-7) cells and human cervical cancer (Hela) cells were cultured in Dulbecco′s Modification of Eagle’s Medium (DMEM), supplemented with 10% (v/v) FBS, 1% penicillin (100 U/mL) and streptomycin (100 μg/mL) at 37 °C in a humidified 5% CO_2-_containing incubator. Animal care and all experimental procedures were performed with the approval of the Animal Care Committee of Fudan University.

### ^99m^Tc tricine-EDDA/HYNIC-SP94 experiments

**^99m^Tc radiolabeling and stability.** Tricine (Sigma-Aldrich Corporation, St Louis, USA) and tricine/EDDA (TCI Corporation, Tokyo, Japan) were selected individually as coligands for ^99m^Tc-labeled HYNIC-SP94. For tricine, the labeling procedure was as briefly follows^24^,^31^,^32^,^33^: in a 1.5 mL test tube, 25 μg HYNIC-SP94 (China Peptides Co Ltd, Hangzhou, China) (1 mg/mL, in H_2_O) was incubated with 200 μL tricine solution (100 mg/mL, in 0.1 M citric-acid buffer, pH 5.0), 14.8~148 MBq ^99m^TcO_4_^−^ solution and 5 μL SnCl_2_ solution (1 mg/mL, in 0.1 M HCl) for 30 min at room temperature. Tricine/EDDA was also used as a coligand^22^,^24^,^34^, as follows: In a 1.5 mL test tube, 2~25 μg of HYNIC-SP94 was incubated with 100 μL of tricine/EDDA solution (20 mg/mL tricine, 10 mg/mL EDDA, pH 6~7), 14.8~148 MBq ^99m^TcO_4_^−^ solution and 5 μL of SnCl_2_ solution for 30 min at room temperature. Following labeling, RCP analysis was performed by instant thin-layer chromatography on silica gel (ITLC-SG) using two systems: 0.1 M citric-acid buffer (pH 5) for detection of free ^99m^TcO_4_^−^ (R_f_ = 0.7~1.0) and 50% acetonitrile (ACN) for detection of ^99m^Tc-colloid (R_f_ = 0). The R_f_ values of the radiolabeled peptide in citric-acid buffer and 50% ACN system were 0.0 and 0.7~1.0, respectively. RCP (%) was calculated using the following formula: (1 − ^99m^TcO_4_^−^ − ^99m^Tc-colloid) × 100%.

The *in vitro* stability of the radiolabeled peptide was tested by incubating it in PBS, saline and FBS for 1, 2, 4, 6 and 12 h at 37 °C. After incubation, ^99m^Tc-labeled complexes were assessed by radio-TLC. Results were plotted as the RCP (%) at different time points.

#### *In vitro* HCC cell competitive receptor binding assay

The binding affinity of HYNIC-SP94 was assessed via a competitive displacement assay with ^99m^Tc tricine-EDDA/HYNIC-SP94^35^. Briefly, Huh-7 cells (1 × 10^6^) and ^99m^Tc tricine-EDDA/HYNIC-SP94 (370 kBq) were added to each test tube in the presence of increasing concentrations of the SP94 peptide (from 0.001 nM to 10000 nM) and incubated for 2 h at 37 °C. To remove unbound ^99m^Tc tricine-EDDA/HYNIC-SP94, the cells were washed three times with cold PBS (1×, pH 7.2) and then centrifuged (600 rpm, 5 min). Radioactivity was determined using a γ-counter. The IC_50_ value was calculated by fitting experimental data with nonlinear regression using GraphPad Prism (GraphPad Software, San Diego, CA, USA).

### Internalization assay

Internalization assay of the ^99m^Tc tricine-EDDA/HYNIC-SP94 was performed according to a previously described method with minor modifications^22^,^26^,^31^,^34^,^36^. Huh-7 cells were diluted to 1 × 10^6 ^cells/tube (~0.5 mL) and incubated with ^99m^Tc tricine-EDDA/HYNIC-SP94 (370 kBq) in triplicate at 37 °C for 0.5, 1, 2 and 4 h. For blocking experiments, Huh-7 cells were incubated with excess unlabeled peptide (2 μg) 30 min prior to incubation with ^99m^Tc tricine-EDDA/HYNIC-SP94. After incubation, the cells were centrifuged (600 rpm, 5 min) and washed three times with PBS to remove free radiolabeled peptides. Cell surface-bound activity was removed by incubation with acid buffer (50 mM glycine-HCl/100 mM NaCl, pH 2.8) at room temperature for 5 min. The cells were centrifuged, washed with PBS and recentrifuged to determine the internalized radioactivity in a γ-counter. Results were expressed as the percentage of total radioactivity (internalized activity/(surface-bound activity + internalized activity)).

### Preparation of animal models

Four- to six-week-old male athymic nude mice were used for preparation of the xenograft tumor models. Huh-7 and Hela cells were subcutaneously injected at a concentration of 5 × 10^6 ^cells/mouse in the right front flank of the mice. The mice were ready for biodistribution and microSPECT/CT imaging study when the tumors reached an average diameter of 0.8~1.0 cm (3~4 weeks after inoculation). Animal care and experimental procedures were accordant with the Helsinki Declaration and were approved by the institutional animal care committee.

### *In vivo* stability

The *in vivo* stability of ^99m^Tc tricine-EDDA/HYNIC-SP94 was evaluated in balb/c mice. ^99m^Tc tricine-EDDA/HYNIC-SP94 (~30 MBq/mouse) in 0.1 mL of saline was injected via the tail vein. Blood was then taken from the heart 10, 30, 60, 120 and 240 min after injection under anesthesia. Blood was collected in a heparin tube and immediately centrifuged (5000 rpm, 5 min) to obtain plasma. A total of 400 μL methanol/trichloromethane (V/V: 4/1) was added to 400 μL of plasma, fully mixed and centrifuged (5000 rpm, 5 min). RP-HPLC analysis was performed with 20 μL supernatant loading filtered by a 0.22 μm filter. The RP-HPLC was equipped with a Zorbax Eclipse XDB-C_18_ column (4.6 × 150 mm) and the following gradients were used at a flow rate of 1 mL/min: 98% solvent A (0.1% trifluoroacetic in 100% water) and 2% solvent B (0.09% trifluoroacetic in 80% ACN and 20% water): 0~5 min; 2% solvent A and 98% solvent B: 5~10 min; 98% solvent A and 2% solvent B: 10~15 min.

### Biodistribution study

Fifteen Huh-7 tumor-bearing mice were randomly divided into three groups. ^99m^Tc tricine-EDDA/HYNIC-SP94 (~0.74 MBq/mouse) in 0.1 mL of saline was injected via tail vein. At 0.5 and 1.5 h p.i., the mice were anesthetized via 2% isoflurane and sacrificed by cervical dislocation. Immediately after sacrifice, the blood, tumors and organs of interest (heart, lungs, liver, spleen, stomach, small intestine, large intestine, kidneys, muscle and thyroid) were harvested, washed with saline and weighed. Radioactivity was determined using a γ-counter. To determine the specific uptake of ^99m^Tc tricine-EDDA/HYNIC-SP94, 5 mice received an intravenous injection of 200 μg of unlabeled HYNIC-SP94 as a blocking agent 30 min prior to injection of ^99m^Tc tricine-EDDA/HYNIC-SP94 (~0.74 MBq/mouse). The mice were sacrificed 30 min p.i. and the experiments performed as described above. Absorption of organs and tissues was calculated as a percentage of the injected dose per gram of tissue mass (%ID/g).

### SPECT/CT imaging

SPECT/CT images were acquired at 0.5 and 1.5 h after ^99m^Tc tricine-EDDA/HYNIC-SP94 (~37 MBq) administration. For the blocking study, Huh-7 tumor-bearing mice were administered an excessive dose (200 μg/mouse) of non-labeled HYNIC-SP94 0.5 h prior to injection of ^99m^Tc-labeled peptide. Mice were placed in the prone position under 2% isoflurane anesthesia and whole-body imaging was performed. The CT scanning parameters used were as follows: tube voltage, 45 keV; current, 150 μA; frame resolution, 256 × 512; and exposure time, 500 ms/frame. Each scan took about 4 min. SPECT was performed after CT scanning with energy peak, 140 keV; matrix, 256 × 256; window width, 10%; resolution, 1 mm/pixel; and scan time, 40 s/projection, with 24 projections in total. The whole-body scan for each mouse took about 20 minutes. Three-dimensional ordered subset expectation maximization images were reconstructed using HiSPECT (Bioscan, Washington DC, USA).

### ^188^Re-HYNIC-SP94 experiments

**Labeling and stability.** The labeling of ^188^Re-HYNIC-SP94 was carried out as previously described^16^,^28^,^30^. Briefly, 50 μL of GH (100 mg/mL) and 10~80 μL of SnCl_2_ solution (10 mg/mL) were reacted with ^188^Re-perrhenate solution (37 MBq) for 1 h at room temperature. An amount of 50 μg or 200 μg HYNIC-SP94 (2 mg/mL) peptide was added to the solution and incubated for 1 h at room temperature. The RCP of ^188^Re-HYNIC-SP94 was determined by radio-TLC. The analytical system uses chromatography paper as the stationary phase and normal saline separated the ^188^ReO_4_^−^ (R_f_ = 0.7~1.0) from ^188^Re-HYNIC-SP94 and colloidal ^188^Re (R_f_ = 0). ITLC-SG was used as the stationary phase and a solution of ethanol: ammonia: water (2:1:5) separated the colloidal ^188^Re (R_f_ = 0) from ^188^Re-HYNIC-SP94 and ^188^ReO_4_^−^ (R_f_ = 0.7~1.0). If necessary, the ^188^Re-HYNIC-SP94 was purified on a Sep-Pak C_18_ column^30^. The column was activated with 10 mL methanol followed by equilibration with 3 mL 0.125 M NH_4_OAc. The sample was then loaded onto the column and washed with 10 mL 0.125 M NH_4_OAc, then the radiolabeled HYNIC-SP94 was eluted with methanol. Finally, the methanol was removed by evaporation under a nitrogen stream and the desired product was redissolved in saline.

The *in vitro* stability of ^188^Re-HYNIC-SP94 was evaluated by incubating ^188^Re-HYNIC-SP94 in normal saline and FBS at room temperature. RCP was analyzed by radio-TLC at 1, 4, 8, 18, 26 and 48 h after incubation to determine the stability.

#### ^188^Re-HYNIC-SP94 cell study and TUNEL assay

Huh-7 and Hela cells were cultured in 24-well plates (0.5 × 10^5 ^cells/well) 2 days before treatment. To assess ^188^Re-HYNIC**-**SP94-mediated apoptosis, cells were co-incubated with three different doses (0.37, 1.11 and 2.22 MBq/mL) of ^188^Re-HYNIC-SP94 in 1 mL cell culture medium in triplicate. Three control studies were performed, with all procedures the same except ^188^Re-HYNIC-SP94 was replaced by medium alone, unlabeled SP94 (2 μg/mL) and ^188^Re isotope (2.22 MBq/mL) in each group. To further evaluate cell apoptosis induced specifically by ^188^Re-HYNIC-SP94 targeting to its receptors, Hela cell was used as an additional control. The above samples were then incubated in a 5% CO_2_ incubator for 15 and 45 h at 37 °C. Cell apoptosis was evaluated using the TUNEL assay with a kit, according to the manufacturer’s instructions^28^,^37^.

#### MicroSPECT/CT imaging for ^188^Re-HYNIC-SP94 therapy evaluation

^99m^Tc tricine-EDDA/HYNIC-SP94 microSPECT/CT imaging was used to evaluate therapeutic efficacy. Images were acquired in the same Huh-7 tumor-bearing mice before and 24 h after injection of ^188^Re-HYNIC-SP94 (~37 MBq). Image protocol was as described above.

#### H&E staining and immunohistochemistry

To evaluate whether there was a visceral injury, slides of lungs, liver and kidneys were stained with H&E. Tumors and indicated organs were collected and fixed in 4% paraformaldehyde, embedded in paraffin. Routine H&E staining and immunohisto-chemistry for CD34, bax and Ki67 were carried out according to the manufacturer’s instructions. Afterwards, slides were viewed with a light microscope and the images were captured.

### Statistical analysis

All experimental data were represented as mean ± standard deviation (SD). Statistical analysis was performed using Student’s t-test and any results with a P-value < 0.05 were considered as statistically significant. The statistical analysis was performed with SPSS 20.0 (IBM, Chicago, Illinois, USA).

## Additional Information

**How to cite this article**: Li, Y. *et al*. Investigation of SP94 Peptide as a Specific Probe for Hepatocellular Carcinoma Imaging and Therapy. *Sci. Rep.* **6**, 33511; doi: 10.1038/srep33511 (2016).

## Figures and Tables

**Table 1:** Biodistribution of ^99m^Tc tricine-EDDA/HYNIC-SP94 in Huh-7 tumor-bearing mice.

Tissue Organ	0.5 h	1.5 h	0.5 h (blocking)
	(%ID/g ± SD)	(%ID/g ± SD)	(%ID/g ± SD)
Heart	0.54 ± 0.13	0.34 ± 0.03	0.58 ± 0.03
Lung	2.10 ± 0.43	1.45 ± 0.04	2.06 ± 0.18
Liver	2.39 ± 1.55	1.98 ± 0.15	2.07 ± 0.20
Spleen	0.84 ± 0.30	0.67 ± 0.04	1.05 ± 0.27
Stomach	0.85 ± 0.18	0.68 ± 0.09	0.84 ± 0.06
Small intestine	0.97 ± 0.22	0.73 ± 0.07	0.78 ± 0.11
Large intestine	0.70 ± 0.09	0.48 ± 0.02	0.54 ± 0.08
Kidney	7.40 ± 2.66	6.14 ± 0.10	7.58±2.25
Tumor	1.02 ± 0.26	0.74 ± 0.07	0.34 ± 0.09
Muscle	0.30 ± 0.05	0.16 ± 0.02	0.23 ± 0.05
Thyroid	0.47 ± 0.09	0.27 ± 0.03	0.52 ± 0.04
Blood	1.17 ± 0.37	0.49 ± 0.08	0.99 ± 0.12
Tumor/muscle	3.96 ± 0.06	4.55 ± 0.10	1.56 ± 0.06

Blocking (0.5 h) or non-blocking (0.5 and 1.5 h) biodistribution data of ^99m^Tc tricine-EDDA/HYNIC-SP94 in balb/c nude mice bearing Huh-7 tumors are listed in the Table. Data are expressed as percentage of %ID/g of mean ± SD.
